# Design of non-autonomous pH oscillators and the existence of chemical beat phenomenon in a neutralization reaction

**DOI:** 10.1038/s41598-021-90301-8

**Published:** 2021-05-26

**Authors:** Hugh Shearer Lawson, Gábor Holló, Norbert Német, Satoshi Teraji, Hideyuki Nakanishi, Robert Horvath, István Lagzi

**Affiliations:** 1 Department of Physics, Budapest University of Technology and Economics, Budafoki út 8, Budapest, 1111 Hungary; 2 MTA-BME Condensed Matter Physics Research Group, Budapest University of Technology and Economics, Budafoki út 8, Budapest, 1111 Hungary; 3 Department of Macromolecular Science and Engineering, Graduate School of Science and Technology, Kyoto Institute of Technology, Matsugasaki, Kyoto 606-8585 Japan; 4 Nanobiosensorics Group, Institute of Technical Physics and Materials Science, Centre for Energy Research, Konkoly Thege M. u. 29-33, Budapest, 1121 Hungary

**Keywords:** Chemistry, Physics

## Abstract

The beat in physical systems is a transparent and well-understood phenomenon. It may occur in forced oscillatory systems and as a result of the interference of two waves of slightly different frequencies. However, in chemical systems, the realization of the latter type of the beat phenomenon has been lacking. Here we show that a periodic titration of acid and alkaline solutions with each other using programmable syringe pumps in a continuous stirred-tank reactor exhibits the beat phenomenon in the temporal pH oscillation pattern if the time periods of sinusoidal inflow rates of the reagents are slightly different. Interestingly, the frequency of the chemical beat pattern follows the well-known relationship from physics, namely the frequency of the beat is equal to the absolute value of the difference of the two wave frequencies. Based on our strategy, we can design and engineer non-autonomous pH oscillatory systems, in which the characteristics of the temporal oscillations (amplitude, time period) can easily and precisely be controlled by the experimental conditions such as the inflow rates and feed concentrations. The demonstrated phenomena can be exploited in practical applications, we use the non-autonomous pH oscillators to drive the reversible assembly and disassembly of pH-sensitive building blocks (oleic acid and gold nanoparticles), both highly relevant in nanotechnology and biomedical applications.

## Introduction

Chemical oscillators are one of the examples of chemical reaction networks in which the sustainable oscillations of the concentration of one of several chemical species can be achieved by coupling of an autocatalytic reaction to a negative feedback^1–3^. These types of oscillations occur in either closed (e.g., Belousov–Zhabotinsky reaction^4–6^, Briggs–Rauscher reaction^7–9^) or open systems^10–14^. In the open system, realized in continuous stirred-tank reactors (CSTRs), in which the inflow of the reagents and outflow from the reactor are allowed and usually their flow rates are constant in time, offer the possibility to maintain the non-linear chemical systems far from their thermodynamic equilibria. pH oscillators (in which the concentration of the H^+^ ions oscillates in time) fall into this type of oscillators and the existing range of the pH are in between acidic and neutral (e.g., bromate–sulfite oscillator^11,14–16^, hydrogen peroxide–sulfite oscillator^17–19^) or neutral and alkaline (sulfite – formaldehyde–lactone oscillators^20–22^) conditions. Due to their chemical mechanism, no oscillator has been designed and engineered which can oscillate between acidic and alkaline conditions and the greatest pH amplitude that can be achieved is around three pH units (ΔpH ~ 3)^23^. The difficulties to utilize pH oscillators in widely used and multipurpose applications lie in two serious issues. First, the time period and amplitude of the oscillations can be only slightly controlled by the initial concentration of the reagents and inflow rates. Secondly, the reagents (e.g., bromate) and produced intermediate chemical species (radicals) generate harsh and oxidative conditions which rendering impossible any use of pH oscillators to control or drive reaction or assembly processes of chemically sensitive chemical species and building blocks (e.g., biologically relevant species such as enzymes and proteins and thiol-protected nanoparticles).

In various branches of physics, namely mechanics, acoustics, and electronics, the beat phenomenon is a widely known and experienced effect. Applications include the police radar, subjective tones, multiphonics, and Doppler pulse detection of moving blood. The beat phenomenon can be manifested in two ways, in forced systems (e.g., forced mechanical oscillators) and in systems in which two harmonic waves of slightly different frequencies overlap and create a new resultant wave (e.g., acoustics)^24^. In forced physical and chemical oscillators, when the frequency of the periodic forcing is close to the natural frequency of the systems, the beat phenomenon emerges, i.e., the envelopes of the oscillation curve have periodic character^25^. In acoustics, a beat is an interference pattern between two sounds of slightly different frequencies. The frequency of the beat ($f_{{\text{b}}} )$ can be calculated from the frequencies ($f_{1}$, $f_{2}$) of the two waves as $f_{{\text{b}}} = \left| {f_{2} - f_{1} } \right|$^24^. The transparent experimental demonstration of the beat phenomenon in chemistry is challenging because it requires that the two frequencies are only slightly different, $f_{{\text{b}}} \ll \left| {f_{2} + f_{1} } \right|$. Therefore, stable and well controlled systems with fine-tuned fundamental frequencies are needed for the beat phenomenon to emerge and being observable at larger time-scales.

To overcome the drawbacks of the autonomous chemical oscillators, here we present a powerful and versatile approach to design and maintain pH oscillations with a desired amplitude and time period in a non-autonomous setup by using a neutralization (acid–base) reaction executed in the CSTR. The chief idea in our setup is to use various periodic time-dependent inflow rate functions (waveforms) of the reagents (acid and alkaline solutions) with an identical time period and the phase difference (*φ*) of π. We also show that if the frequency (time period) of the inflow rate functions of the acidic and alkaline solutions differs from each other, the beat phenomenon emerges in the pH oscillations. Interestingly, the frequency of the beat of the neutralization reaction in the CSTR follows the well-known relation from physics, namely the beat frequency is equal to the absolute value of the difference of the frequencies of two inflow rate wave functions.

## Results

**Figure 1:**
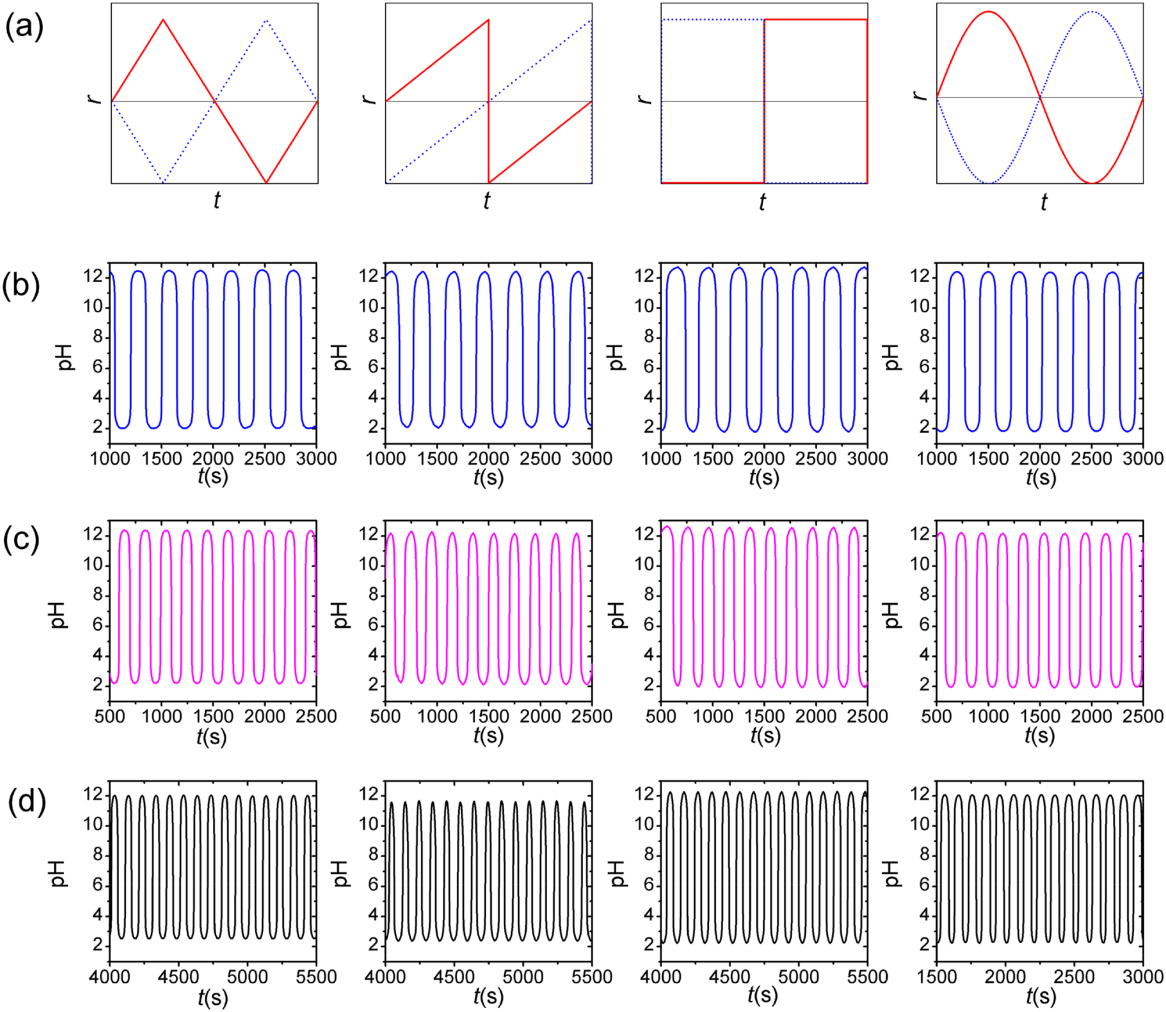
Non-autonomous pH oscillations in the acid–base neutralization reaction by using various time-dependent inflow rate functions (waveforms) of the reagents, acid and alkaline solutions, with the phase difference of π (**a**). The time periods of the various inflow rate functions (triangular, sawtooth, square and sinusoidal) were identical, $T_{{{\text{acid}}}} = T_{{{\text{base}}}} = 300\, {\text{s}}$ (**b**), $T_{{{\text{acid}}}} = T_{{{\text{base}}}} = 200\, {\text{s}}$ (**c**), and $T_{{{\text{acid}}}} = T_{{{\text{base}}}} = 100 \,{\text{s}}$ (**d**). The concentrations of the acid and alkaline solutions in the input feed were 0.1 M ($c_{{{\text{H}}^{ + } }}^{0} = c_{{{\text{OH}}^{ - } }}^{0} = 0.1 \,{\text{M}})$, respectively.

To gain more insight into the capability of our approach, first, we designed and generated pH oscillations by using various periodic time-dependent inflow rate functions (waveforms) of the reagents (acid and alkaline solutions) in a way that the time periods were identical (set to *T* = 300 s, 200 s, and 100 s) with the phase difference of π between the two inflow rate functions. We used and tested four mostly widespread waveform functions used in physics and engineering such as triangular, sawtooth, square, and sinusoidal waveforms of the inflow rates of acid and alkaline solutions. Figure 1 presents the applied waveforms of the inflow rates and the generated pH oscillations. Due to the phase difference of π between the inflow rates functions, the existence of the pH oscillations can be simply explained by the periodic change of the excess of either hydrogen or hydroxide ions in the CSTR. The generated oscillations have similar characteristics, namely the peak-to-peak amplitude, the time period, and the residence time at lower and higher pH. A trivial consequence of the experimental design is that the non-autonomous oscillatory system emulates the time period of the periodic inflow rate functions. The time spent by the system at the acidic region is identical to that of the alkaline condition within the experimental error (triangular waveform: 51 ± 1.8%, sawtooth waveform: 48 ± 2.6%, square waveform: 50 ± 3.1%, sinusoidal waveform: 52 ± 0.67%).

When the time period of the periodic inflow rate functions decreased, the peak-to-peak amplitude of the oscillations slightly decreased (Fig. 1). Numerical and analytical calculations show similar oscillations behavior observed in experiments (Supplementary Fig. S1).

**Figure 2:**
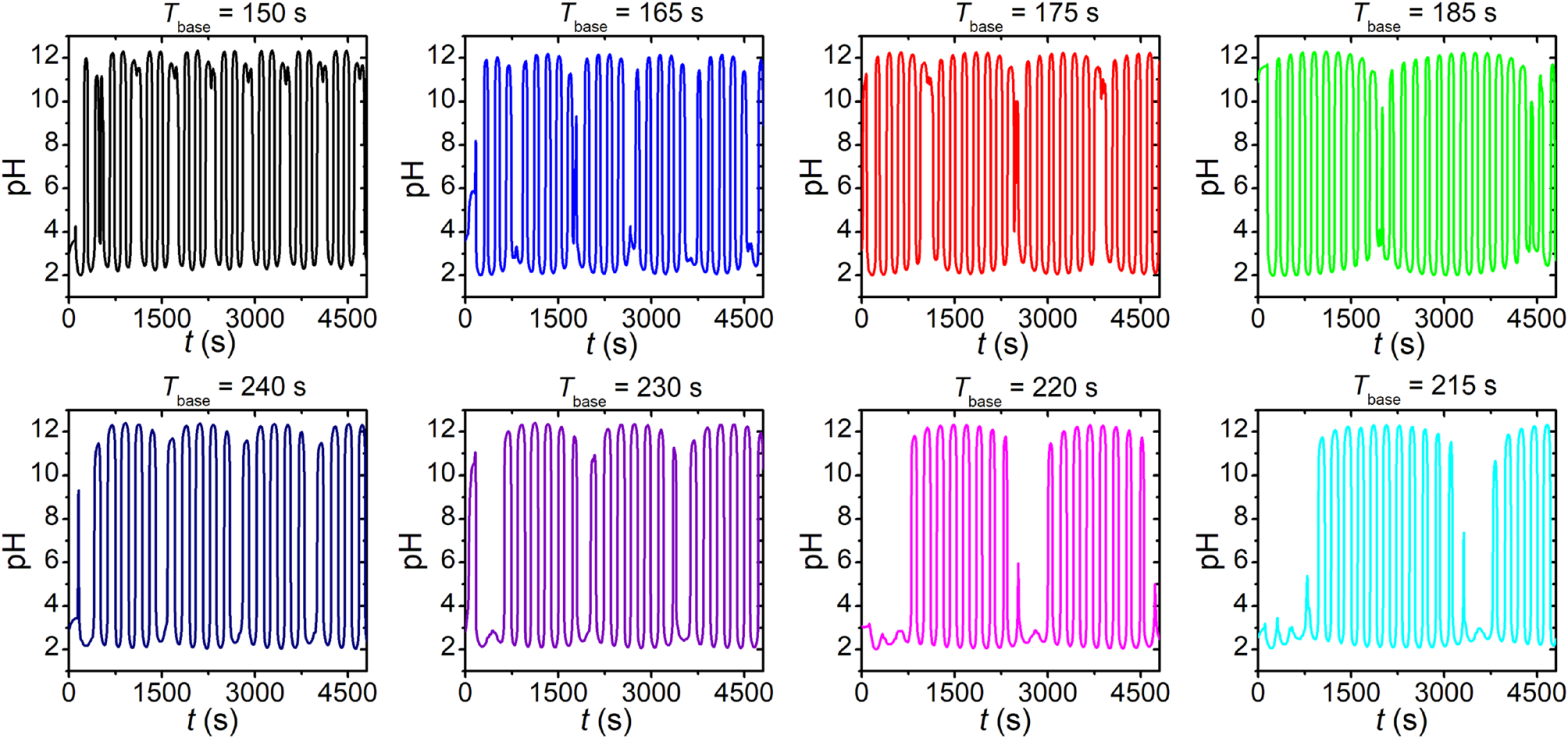
Beat phenomenon in the acid–base neutralization reaction using sinusoidal time-dependent inflow rate functions of the reagents, acid $\left( {r\left( t \right) = { }r_{0} + r_{{\text{A}}} {\text{sin}}\left( {\frac{2\pi }{{T_{{{\text{acid}}}} }}t} \right)} \right)$ and alkaline $\left( {r\left( t \right) = { }r_{0} + r_{{\text{A}}} {\text{sin}}\left( {\frac{2\pi }{{T_{{{\text{base}}}} }}t + \varphi } \right)} \right)$ solutions with $\varphi$ = π, ${ }r_{0}$ and ${ }r_{{\text{A}}}$ were 15 µL s^−1^. The time period of the inflow rate of the acid was fixed, $T_{{{\text{acid}}}} = 200\; {\text{s}}$, and in each experiment $T_{{{\text{base}}}}$ was varied. The concentrations of the acid and alkaline solutions in the input feed were 0.1 M ($c_{{{\text{H}}^{ + } }}^{0} = c_{{{\text{OH}}^{ - } }}^{0} = 0.1 {\text{M}})$, respectively.

**Figure 3:**
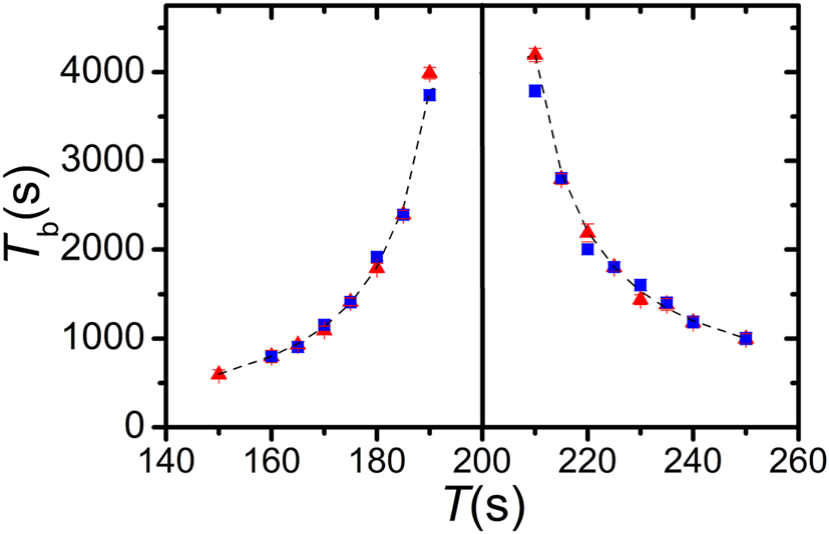
Dependence of the time period of the chemical beat phenomenon ($T_{{\text{b}}}$) on the time period of one inflow rate (the time period of other inflow rate was fixed to 200 s) observed in the experiments in the acid–base neutralization reaction using sinusoidal time-dependent inflow rate functions of the reagents, acid $\left( {r\left( t \right) = { }r_{0} + r_{{\text{A}}} {\text{sin}}\left( {\frac{2\pi }{{T_{{{\text{acid}}}} }}t} \right)} \right)$ and alkaline $\left( {r\left( t \right) = { }r_{0} + r_{{\text{A}}} {\text{sin}}\left( {\frac{2\pi }{{T_{{{\text{base}}}} }}t + \varphi } \right)} \right)$ solutions with $\varphi$ = π, ${ }r_{0}$ and ${ }r_{{\text{A}}}$ were 15 µL s^−1^. Blue and red symbols present the data when the time period of acid and base inflow rates was fixed (*T* = 200 s) once the time period of base and acid inflow rates was changed in the experiments, respectively. The concentrations of the acid and alkaline solutions in the input feed were 0.1 M ($c_{{{\text{H}}^{ + } }}^{0} = c_{{{\text{OH}}^{ - } }}^{0} = 0.1 {\text{M}})$, respectively. The dashed lines represent the theoretically calculated curves from the relation $T_{{\text{b}}} = \left| {1/\left( {\left( {T_{{{\text{acid}}}} } \right)^{ - 1} - \left( {T_{{{\text{base}}}} } \right)^{ - 1} } \right)} \right|$, i.e., $f_{{\text{b}}} = \left| {f_{{{\text{acid}}}} - f_{{{\text{base}}}} } \right|$.

After exploring the basics features of the non-autonomous oscillators, we intended to investigate the oscillation pattern when the inflow rate functions have different time periods with a phase difference of π. In our experiments, we used the sinusoidal modulation for the inflow rates because it is the most common waveform in physics exhibiting the beat phenomenon. In the first set of experiments, we carried out investigations fixing the time period of the inflow rate of the acid solution ($T_{{{\text{acid}}}} = 200 \;{\text{s}}$) and varying the time period of the inflow rate of the alkaline solution. Figure 2 and Supplementary Fig. S2 present vividly the appearance and existence of the beat phenomenon in the temporal oscillation pattern. Most importantly, the time period/frequency of the beat follows the common relationship valid and known for various types of physical waves and oscillators, i.e., $f_{{\text{b}}} = \left| {f_{{{\text{acid}}}} - f_{{{\text{base}}}} } \right|$, expressing by the time periods $T_{{\text{b}}} = \left| {1/\left( {\left( {T_{{{\text{acid}}}} } \right)^{ - 1} - \left( {T_{{{\text{base}}}} } \right)^{ - 1} } \right)} \right|$ (Fig. 3). We also carried out experiments in a reverse way, namely this time the time period of the inflow rate of the alkaline solution was fixed ($T_{{{\text{base}}}} = 200\; {\text{s}}$) and the time period of the inflow rate of the acid solution was changed. In this case, we obtained the same beat frequency within the experimental error limits (Fig. 3). The numerical and analytical models reproduce the experimentally observed beat phenomenon (Supplementary Fig. S3).

**Figure 4:**
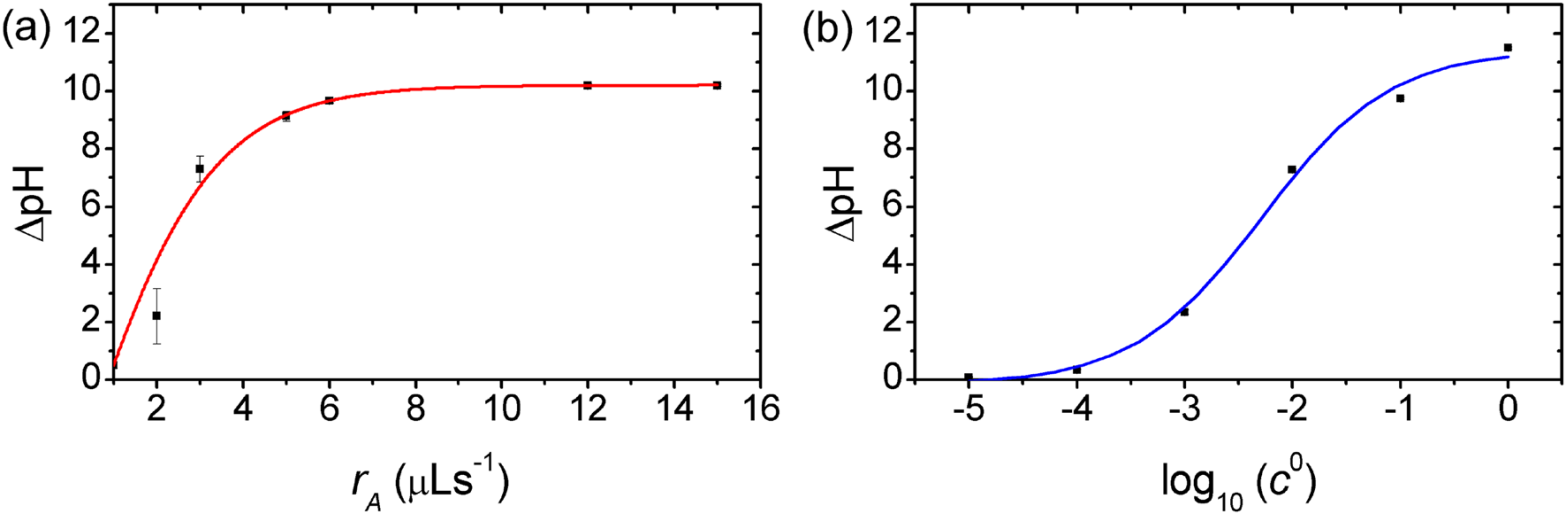
Control of the amplitude of the pH oscillations. Dependence of the peak-to-peak amplitude of the oscillations (ΔpH) on (**a**) $r_{{\text{A}}}$, amplitude of the sinusoidal rate functions (${ }r_{0}$ was 15 µL s^−1^), and (**b**) *c*, the concentrations of the acid and alkaline solutions in the input feed $\left( {c^{0} = c_{{{\text{H}}^{ + } }}^{0} = c_{{{\text{OH}}^{ - } }}^{0} } \right)$, using sinusoidal time-dependent inflow rate functions of the reagents, acid $\left( {r\left( t \right) = { }r_{0} + r_{{\text{A}}} {\text{sin}}\left( {\frac{2\pi }{{T_{{{\text{acid}}}} }}t} \right)} \right)$ and alkaline $\left( {r\left( t \right) = { }r_{0} + r_{{\text{A}}} {\text{sin}}\left( {\frac{2\pi }{{T_{{{\text{base}}}} }}t + \varphi } \right)} \right)$ solutions with $\varphi$ = π.

In design procedures of oscillatory systems, it is a key challenge how the characteristics of the oscillations can be fine-tuned by the experimental setup and conditions (such as the temperature, the initial concentration of the reagents, the inflow rate). In autonomous oscillatory systems, these parameters and conditions provide only a limited degree of freedom in adjusting the oscillation behavior of the chemical system. In our system, there is an intuitive idea to adjust the peak-to-peak amplitude of the oscillations (ΔpH) by the amplitude of the sinusoidal inflow rates ($r_{{\text{A}}}$) and the concentrations of the reagents in the input feed ($c_{{{\text{H}}^{ + } }}^{0}$, $c_{{{\text{OH}}^{ - } }}^{0}$). When the amplitude of the sinusoidal inflow rates decreased from 15 µL s^−1^ (which is the maximum value since ${ }r_{0}$ was 15 µL s^−1^ in the experiments) to 5 µL s^−1^ (decreased by 2/3rd), ΔpH did not change significantly (Fig. 4a). However, when it was decreased to 2 µL s^−1^, the peak-to-peak amplitude of the oscillations decreased exponentially from ΔpH ~ 9 to ΔpH ~ 2. Similar behavior can be observed, if the concentrations of the acid and alkaline solutions in the input feed were decreased from 1 M to 10^−5^ M (Fig. 4b). As it can be expected, decreasing the concentrations in the input feed generated gradually decreasing of ΔpH, and it practically went to zero at 10^−5^ M.

In the design procedure of pH oscillators, one of the key features is the ratio of time spent by the system at higher and lower pH states. In autonomous oscillators, the feature of the temporal pH oscillation pattern depends rather on the mechanism of the system than the experimental conditions (e.g., the concentration of the reagents, the temperature, and the inflow rates). Therefore, the profiles of the oscillations can hardly be controlled by the experimental parameters. However, using our non-autonomous approach by changing the phase difference (*φ*) between the inflow rates of the acid and alkaline solutions provides a versatile way to control this ratio. When the *φ* was varied from π/4 to π, the time spent in the acidic range by the oscillator changed from 70 to 50% (Supplementary Fig. S4), this observation is in good accordance with the results obtained from the numerical model simulations (Supplementary Fig. S5).

**Figure 5:**
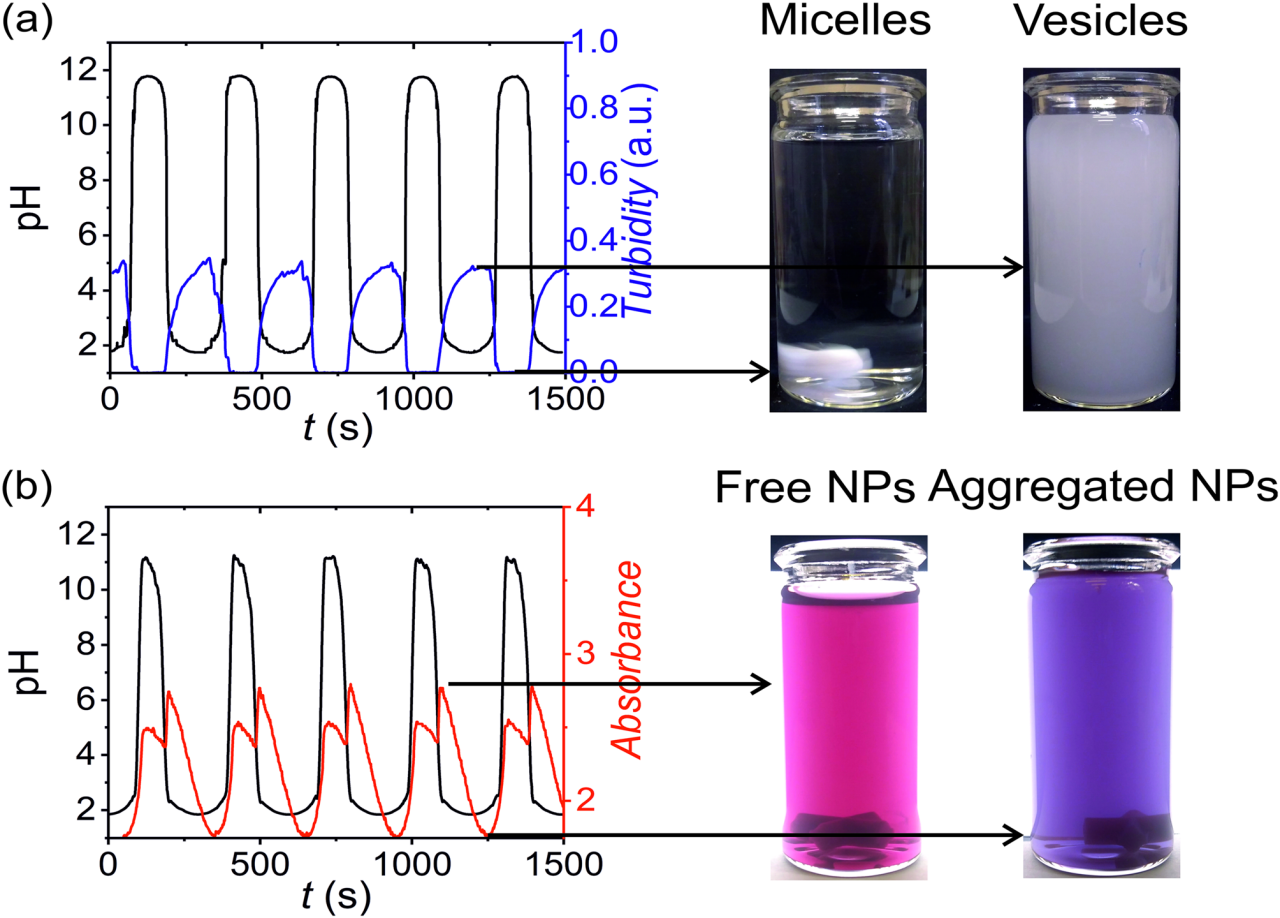
Reversible transformation of vesicle and micelles (**a**) and assembly/disassembly of pH-sensitive AuNPs (**b**) using the non-autonomous pH oscillator (sinusoidal function, $\varphi$ = π, $T_{{{\text{acid}}}} = T_{{{\text{base}}}} = 300 \,{\text{s}}$). The concentrations of the acid and alkaline solutions in the input feed were 0.1 M ($c_{{{\text{H}}^{ + } }}^{0} = c_{{{\text{OH}}^{ - } }}^{0} = 0.1\, {\text{M}})$, respectively.

To illustrate the applicability of our approach, we controlled the reversible vesicle/micelle transition of oleic acid molecules^26^ and assembly/disassembly of pH-sensitive gold nanoparticles (AuNPs) by using the non-autonomous pH oscillator^27^. Figure 5 summarizes the results of these investigations. First, when we used fatty acid molecules, once the pH reached the very alkaline state (pH > 10), all oleic acid molecules were deprotonated forming micellar (transparent) solution. However, when pH started to drop more molecules became protonated and formed fatty acid vesicles and emulsion at pH close to the pKa of the oleic acid and below, respectively, forming a white turbid phase in the solution^28^. The periodic pH change generated vesicle/micelle transformation, which was manifested in a periodic change in the turbidity of the solution (Fig. 5a). In the case of using AuNPs, when the system was at the low‐pH state, carboxyl-terminated thiol ligands (attached to the surface of the AuNPs) were protonated (uncharged). Therefore, the electrostatic repulsions between the AuNPs were weak, and the interparticle interactions were predominantly realized by van der Waals (vdW) attractions causing aggregation of the AuNPs. When the pH oscillator reached a high pH state, the ligands become deprotonated (charged) and electrostatic repulsions between the particles caused the particles to disperse. The color of the solution of AuNPs depends on their aggregation state and originates due to the surface plasmon resonance (SPR). The solution of AuNPs comprising a few nanometers unaggregated particles has a red color ($\lambda_{{{\text{max}}}} \sim 520 \,{\text{nm}}$), when these AuNPs aggregate forming clusters, the color of the solution changes from red to blue, which causes an absorbance decrease at $\lambda_{{{\text{max}}}}$. In other words, the pH oscillations translated into the rhythmic aggregation/dispersion of the AuNPs, which generated color oscillation of the solution and absorbance oscillation at a fixed wavelength (Fig. 5b).

## Discussion

In this study, we introduced a new concept in designing pH oscillators of arbitrary time period and amplitude. We followed a freshly new approach, namely instead of exploring and discovering new components of the autonomous reaction networks and combining them to generate oscillations, we simply titrated an acid solution with an alkaline solution and vice versa in a CSTR by using periodic inflow rate functions of the reagents with a phase difference of π to maintain pH oscillations. By varying the amplitude of the periodic inflow rate functions and the concentrations of the acid and alkaline solutions in the input feed, the amplitude of the pH oscillations can be easily adjusted to the desired value. In addition, by adjusting the phase difference between the inflow rates of the acid and alkaline solutions, the time spent in the acidic range by the oscillator can be fine-tuned within 40%. A trivial consequence of this strategy is that by increasing the concentration of either acid or alkaline solution and/or inflow rates of the reagents, oscillations occurring solely at either acidic or alkaline pH range can be obtained. Similarly, the behavior of the system can be adjusted by replacing the strong acid with the weak one and periodically titrated by the strong base or vice versa. As we have shown, our method could be a perfect candidate to control the pH-induced assembly and disassembly of pH-sensitive building blocks (e.g., nanoparticles, proteins, polyelectrolyte multilayers). These non-autonomous pH oscillators could be successfully utilized in the studies and applications, in which bio- and chemically sensitive chemical compounds are involved because autonomous oscillators usually use oxidative chemical species and generate free radicals as intermediate species.

The main scientific message of this study that we transparently presented a chemical system in which the beat phenomenon emerges in the pH oscillation pattern when the time periods of the inflow rate functions of acid and alkaline solutions differ. This chemical beat phenomenon is a chemical counterpart of the well-known and studied beat phenomena in physical systems, when two longitudinal or transversal waves with different frequencies generate the beat pattern. Interestingly, we found that the beat frequency follows the classical dependence on the two frequencies of the interfering waves known in physics. This is the simplest chemical system comprising only a neutralization reaction which is capable of exhibiting complex behaviors such as pH oscillations and beat phenomenon under special circumstances in a non-autonomous setup.

## Methods

### Experimental setup

In our experiments, the acid–base neutralization reaction was carried out in a continuous stirred-tank reactor (CSTR) with a volume of 7.0 mL and a stirring rate of 800 rpm at 22.0 ± 0.5 °C. We used the following reagent-grade chemicals, hydrochloric acid (HCl, Sigma-Aldrich), sodium hydroxide (NaOH, Sigma-Aldrich) and potassium nitrate (KNO_3_, Sigma-Aldrich). Potassium nitrate was used as an inert salt in experiments, in which the effect of the concentrations of acid and alkaline solutions on the amplitude of the pH oscillation was investigated. Two solutions of acid and base with identical concentrations were allowed to flow simultaneously with the modulated flow rate into the reactor using two programmable syringe pumps. Four types of inflow rate functions were used: triangular, sawtooth, square, and sinusoidal waves. The corresponding functions were the following (Fig. 1a):

1 $$ {\text{triangular}}\,{\text{ function}}:r\left( t \right) = \left\{ {\begin{array}{*{20}c} {\begin{array}{*{20}c} {\frac{{r_{{{\text{max}}}} - r_{{{\text{min}}}} }}{2} + \frac{{r_{{{\text{max}}}} - r_{{{\text{min}}}} }}{T/2}t, {\text{if}} \;0 \le t < \frac{T}{4}} \\ {\left( {r_{{{\text{max}}}} + \frac{{r_{{{\text{max}}}} - r_{{{\text{min}}}} }}{2}} \right) - \frac{{r_{{{\text{max}}}} - r_{{{\text{min}}}} }}{T/2}t, {\text{if}}\;\frac{T}{4} \le t < \frac{3T}{4}} \\ \end{array} } \\ {\left( {r_{{{\text{min}}}} - r_{{{\text{max}}}} - \frac{{r_{{{\text{max}}}} - r_{{{\text{min}}}} }}{2}} \right) + \frac{{r_{{{\text{max}}}} - r_{{{\text{min}}}} }}{T/2}t,{\text{if}}\;\frac{3T}{4} \le t< T,} \\ \end{array} } \right. $$

2 $$ {\text{sawtooth}}\,{\text{ function}}:r\left( t \right) = \left\{ {\begin{array}{*{20}c} {\frac{{r_{{{\text{max}}}} - r_{{{\text{min}}}} }}{2} + \frac{{r_{{{\text{max}}}} - r_{{{\text{min}}}} }}{T}t, {\text{if}} \;0 \le t < T/2} \\ { - r_{{{\text{max}}}} + \frac{{r_{{{\text{max}}}} - r_{{{\text{min}}}} }}{T}t, {\text{if}} \;T/2 \le t  <T,} \\ \end{array} } \right. $$

3 $$ {\text{square }}\,{\text{function}}:r\left( t \right) = \left\{ {\begin{array}{*{20}c} {{ }r_{{{\text{max}}}} , {\text{if}}\;0 \le t < T/2} \\ {r_{{{\text{min}}}} , {\text{if}}\;T/2 \le t < T,} \\ \end{array} } \right. $$

4 $$ {\text{sinusoidal}}\,{\text{ function}}:r\left( t \right) = { }r_{0} + r_{{\text{A}}} {\text{sin}}\left( {\frac{2\pi }{T}t} \right), $$

where *T* is the time period of the waves. $r_{{{\text{max}}}}$ and $r_{{{\text{min}}}}$ are the maximum and minimum flow rates, in our study ${ }r_{{{\text{max}}}}$ and ${ }r_{{{\text{min}}}}$ were set to 30 µL s^−1^ and 0, respectively. ${ }r_{0}$ was 15 µL s^−1^. In a typical experiments, the phase difference of *φ* = π was applied between the inflow rate functions of acid and base. In beat experiments, the phase difference was similarly set to π, the various time periods were applied for the inflow rate functions for acid and alkaline solutions. The pH in CSTR was monitored by a pH microelectrode (Mettler Toledo Lab pH Electrode LE422). In experiments, in which lower concentrations of acid and alkaline solutions than 0.1 M were deployed, an inner conducting salt (KNO_3_, Sigma-Aldrich) was used with the concentration of 0.1 M to maintain the proper ionic strength for measuring the pH with a glass electrode.

In the investigation of the reversible micelle-vesicles transformation of the oleic acid and assembly/disassembly of AuNPs and, we used oleic acid (Sigma-Aldrich), and carboxyl-terminated thiol (mercaptoundecanoic acid, MUA, Sigma-Aldrich) stabilized AuNPs synthesized by a modified procedure^29^. The average size of the particles was 4.4 nm with a dispersity (polydispersity index) of 0.09 (Supplementary Fig. S6). The oleic acid and AuNPs were initially dispersed in a stock solution with concentrations of 4.75 mM (pH = 11) and 6.5 mM (in terms of gold atoms, pH = 11), respectively. The acid–base neutralization reaction was carried out in a CSTR (cylindrical glass cuvette of 2.2 cm optical path length) with a volume of 10.0 mL and a stirring rate of 800 rpm at 22.0 ± 0.5 °C. To generate the pH oscillations, we used a sinusoidal modulation (${ }r_{0}$ and ${ }r_{{\text{A}}}$ were 12.0  µL s^−1^) with the phase difference of *φ* = π and a time period of 300 s. The solution of the oleic acid and AuNPs were introduced into the CSTR with a constant flow rate of 2.0 µL s^−1^. We followed the processes in time by using UV–Vis spectrophotometry in kinetic mode (UV-1600PC spectrophotometer) simultaneously measuring the pH with a microelectrode (Mettler Toledo Lab pH Electrode LE422). The wavelengths for UV–Vis kinetic measurements were 600 nm and 533 nm for the oleic acid and AuNPs, respectively. The wavelength of 533 nm is the maximum absorbance for the SPR of small and non-aggregated AuNPs.

### Numerical model

Our numerical model consists of a reversible step for the water formation from the hydrogen and hydroxide ions:

5 $$ {\text{H}}^{ + } + {\text{ OH}}^{ - } \to {\text{H}}_{{2}} {\text{O}}\,{\text{ with}}\,{\text{ the }}\,\,{\text{chemical}}\,{\text{ rate }}\,{\text{constant }}\,{\text{of}}\,k_{{1}} = { 1}0^{{ - {11}}} \left( {{\text{M}}\,{\text{ s}}} \right)^{{ - {1}}} , $$

6 $$ {\text{H}}_{{2}} {\text{O}} \to {\text{H}}^{ + } + {\text{ OH}}^{ - } \,{\text{with}}\,{\text{ the }}\,{\text{chemical }}\,{\text{rate }}\,{\text{constant}}\,{\text{ of}}\,k_{{2}} = { 1}0^{{ - {3}}} \,{\text{M }}\,{\text{s}}^{{ - {1}}} . $$

The chemical system can be described by the following set of differential equations

7 $$ \frac{{{\text{d}}c_{{{\text{H}}^{ + } }} }}{{{\text{d}}t}} = k_{2} - k_{1} c_{{{\text{H}}^{ + } }} c_{{{\text{OH}}^{ - } }} + { }\kappa_{{{\text{H}}^{ + } }} c_{{{\text{H}}^{ + } }}^{0} - \kappa^{*} c_{{{\text{H}}^{ + } }} , $$

8 $$ \frac{{{\text{d}}c_{{{\text{OH}}^{ - } }} }}{{{\text{d}}t}} = k_{2} - k_{1} c_{{{\text{H}}^{ + } }} c_{{{\text{OH}}^{ - } }} + \kappa_{{{\text{OH}}^{ - } }} c_{{{\text{OH}}^{ - } }}^{0} - { }\kappa^{*} c_{{{\text{OH}}^{ - } }} , $$

where $c_{{{\text{H}}^{ + } }}$ and $c_{{{\text{OH}}^{ - } }}$ are concentrations of the hydrogen and hydroxide ions, $k_{1}$ and $k_{2}$ are the chemical rate constants of reactions Eqs. (5, 6). ${ }\kappa_{{{\text{H}}^{ + } }}$ and ${ }\kappa_{{{\text{OH}}^{ - } }}$ are the inflow rates of the hydrogen and hydroxide ions, and ${ }\kappa^{*}$ is the outflow rate of the ions. In the simulations, we considered only sinusoidal inflow rates, and these rates were calculated as follows, ${ }\kappa_{{{\text{H}}^{ + } }} = { }\kappa_{0} + { }\kappa_{{\text{A}}} {\text{sin}}\left( {\frac{2\pi }{{T_{{{\text{acid}}}} }}t} \right)$, ${ }\kappa_{{{\text{OH}}^{ - } }} = { }\kappa_{0} + { }\kappa_{{\text{A}}} {\text{sin}}\left( {\frac{2\pi }{{T_{{{\text{base}}}} }}t + \varphi } \right)$, ${ }\kappa^{*} = { }\kappa_{{{\text{H}}^{ + } }} + { }\kappa_{{{\text{OH}}^{ - } }}$, and ${ }\kappa_{0} = { }\kappa_{{\text{A}}} = 2.143 \times 10^{3}$ s^−1^ (determined from the experimental conditions, $\kappa_{0} = r_{0} /V$, where $V$ is the volume of CSTR. $c_{{{\text{H}}^{ + } }}^{0}$ and $c_{{{\text{OH}}^{ - } }}^{0}$ are the concentrations of the hydrogen and hydroxide ions in the input feed, it was set to $c_{{{\text{H}}^{ + } }}^{0} = c_{{{\text{OH}}^{ - } }}^{0} = 0.1{\text{M}}$. The ordinary differential equations were solved using MATLAB software with the ode15s solver (relative tolerance = 10^−11^, absolute tolerance = 10^−14^). The initial conditions were the following, $c_{{{\text{H}}^{ + } }} \left( {t = 0} \right) = c_{{{\text{OH}}^{ - } }} \left( {t = 0} \right) = 10^{7} {\text{M}}$.

### Analytical model

The analytical model has several reasonable simplifications compared to the numerical model, namely, the neutralization reaction is irreversible (*k*_2_ = 0) and instantaneous (*k*_1_ → ∞), and similarly to the numerical model, we took into account a sinusoidal waveform of the inflow rates of the reagents. We introduce a variable *x*(*t*), which describes the pH of the system,

9 $$ {\text{pH}} = { } - {\text{log}}_{10} x\left( t \right),\, {\text{if }}\,x\left( t \right) > 10^{ - 7} \,{\text{M,}} $$

10 $$ {\text{pH}} = { }7, \,{\text{if }}\,\left| {x\left( t \right)} \right| \le 10^{ - 7} \,{\text{M,}} $$

11 $$ {\text{pH}} = { }14 + {\text{log}}_{10} \;\left| {x\left( t \right)} \right|, \,{\text{if }}\,x\left( t \right) < -10^{ - 7} \,{\text{M}}{.} $$

Based on the assumptions above, the system can be described by the following ordinary differential equation

12 $$ \frac{{{\text{d}}x\left( t \right)}}{{{\text{d}}t}} = \left( {1 + {\text{sin}}\left( {\frac{2\pi }{{T_{{{\text{acid}}}} }}t + \frac{\pi }{2}} \right)} \right)\kappa_{0} c^{0} - \left( {1 + {\text{sin}}\left( {\frac{2\pi }{{T_{{{\text{base}}}} }}t - \frac{\pi }{2}} \right)} \right)\kappa_{0} c^{0} - \left( {\left( {1 + {\text{sin}}\left( {\frac{2\pi }{{T_{{{\text{acid}}}} }}t + \frac{\pi }{2}} \right)} \right)\kappa_{0} + \left( {1 + {\text{sin}}\left( {\frac{2\pi }{{T_{{{\text{base}}}} }}t - \frac{\pi }{2}} \right)} \right)\kappa_{0} } \right)x\left( t \right), $$

where $c^{0} = c_{{{\text{H}}^{ + } }}^{0} = c_{{{\text{OH}}^{ - } }}^{0}$. If $T_{{{\text{acid}}}} = T_{{{\text{base}}}} = T$, i.e., the time periods of the inflow rate functions are identical, the Eq. (12) has the analytical solution of the following form

13 $$ x\left( t \right) = \frac{{\pi \kappa_{0} c^{0} T{\text{sin}}\left( {\frac{2\pi }{T}t} \right)}}{{\kappa_{0}^{2} T^{2} + \pi^{2} }} + \frac{{c^{0} \kappa_{0}^{2} T^{2} {\text{cos}}\left( {\frac{2\pi }{T}t} \right)}}{{\kappa_{0}^{2} T^{2} + \pi^{2} }} + \alpha e^{{ - 2{ }\kappa_{0} t}} , $$

where $\alpha$ was set to fulfill the initial condition of $x\left( {t = 0} \right) = 0.$

If different time periods were used ($T_{{{\text{acid}}}} \ne T_{{{\text{base}}}}$), the Eq. (12) has the analytical solution of

14 $$ \begin{aligned} x\left( t \right) & = {\text{exp}}\left( {\frac{{\kappa_{0} T_{{{\text{acid}}}} {\text{sin}}\left( {\frac{2\pi }{{T_{{{\text{acid}}}} }}t} \right)}}{2\pi } + \frac{{\kappa_{0} T_{{{\text{base}}}} {\text{sin}}\left( {\frac{2\pi }{{T_{{{\text{base}}}} }}t} \right)}}{2\pi } - 2\kappa_{0} t} \right) \\ & \quad \times \mathop \smallint \limits_{1}^{t} \left\{ {\exp \left( {2\kappa_{0} \xi + \frac{{\kappa_{0} T_{{{\text{acid}}}} {\text{sin}}\left( {\frac{2\pi }{{T_{{{\text{acid}}}} }}\xi } \right)}}{2\pi } - \frac{{\kappa_{0} T_{{{\text{base}}}} {\text{sin}}\left( {\frac{2\pi }{{T_{{{\text{base}}}} }}\xi } \right)}}{2\pi }} \right)\left( {\kappa_{0} c^{0} {\text{cos}}\left( {\frac{2\pi }{{T_{{{\text{acid}}}} }}\xi } \right) + \kappa_{0} c^{0} {\text{cos}}\left( {\frac{2\pi }{{T_{{{\text{base}}}} }}\xi } \right)} \right)} \right\}d\xi \\ & \quad + \alpha \exp \left( {\frac{{\kappa_{0} T_{{{\text{acid}}}} {\text{sin}}\left( {\frac{2\pi }{{T_{{{\text{acid}}}} }}t} \right)}}{2\pi } + \frac{{\kappa_{0} T_{{{\text{base}}}} {\text{sin}}\left( {\frac{2\pi }{{T_{{{\text{base}}}} }}t} \right)}}{2\pi } - 2\kappa_{0} t} \right) \\ \end{aligned} $$

The integral in the analytical solution (Eq. 14) was numerically calculated by using MATLAB^30^.

## Supplementary Information

Supplementary Information.
