# Supplementary material for: Design of non-autonomous pH oscillators and the existence of chemical beat phenomenon in a neutralization reaction
Source: Sci Rep. 2021 May 26;11:11011. doi: 10.1038/s41598-021-90301-8 (PMC8155044; doi:10.1038/s41598-021-90301-8)
Supplement: Supplementary file 1 — Supplementary Information. [file 41598_2021_90301_MOESM1_ESM.pdf]

Supplementary Information

Design of non-autonomous pH oscillators and the existence of chemical beat phenomenon in a neutralization reaction

Hugh Shearer Lawson[a], *Gábor Holló*[b], *Norbert Német*[a], *Satoshi Teraji*[c], *Hideyuki Nakanishi*[c], *Robert Horvath*[d], *István Lagzi*[a,b]*

[a]Department of Physics, Budapest University of Technology and Economics, H-1111 Budafoki út 8, Budapest, Hungary

[b]MTA-BME Condensed Matter Physics Research Group, Budapest University of Technology and Economics, H-1111 Budafoki út 8, Budapest, Hungary

[c]Department of Macromolecular Science and Engineering, Graduate School of Science and Technology, Kyoto Institute of Technology, Matsugasaki, Kyoto 606-8585, Japan

[d]Nanobiosensorics Group, Institute of Technical Physics and Materials Science, Centre for Energy Research, H-1121 Konkoly Thege M. u. 29-33, Budapest, Hungary

*E-mail: istvanlagzi@gmail.com

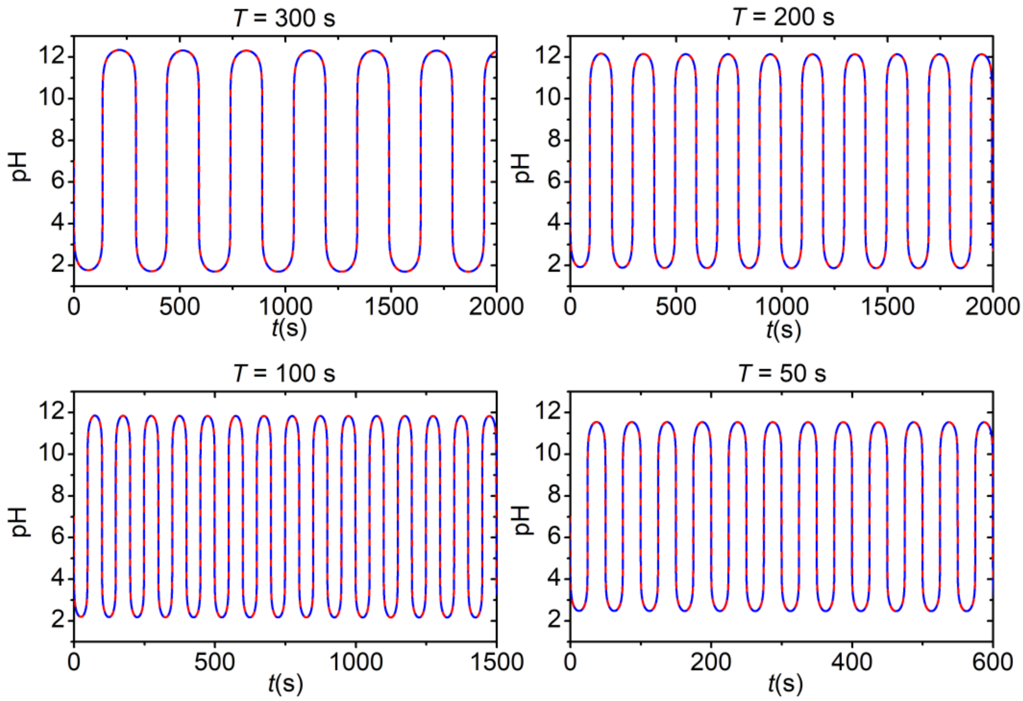

Supplementary Fig. 1 Beat phenomenon in the acid-base neutralization reaction using sinusoidal time-dependent inflow rate functions of the reagents in analytical (solid blue line) and numerical (dashed red line) model, acid $\kappa_{H^+} = \kappa_0 + \kappa_A \sin\left(\frac{2\pi}{T_{acid}} t\right)$, and alkaline $\kappa_{OH^-} = \kappa_0 + \kappa_A \sin\left(\frac{2\pi}{T_{base}} t + \varphi\right)$ solutions with $\varphi = \pi, \kappa_0 = \kappa_A = 2.143 \times 10^{-3}$. The time period of the inflow rates of the acid and alkaline solutions were fixed, $T_{acid} = T_{base} = T$. The concentrations of the acid and alkaline solutions in the input feed were 0.1 M ($c^0_{H^+} = c^0_{OH^-} = 0.1$ M), respectively.

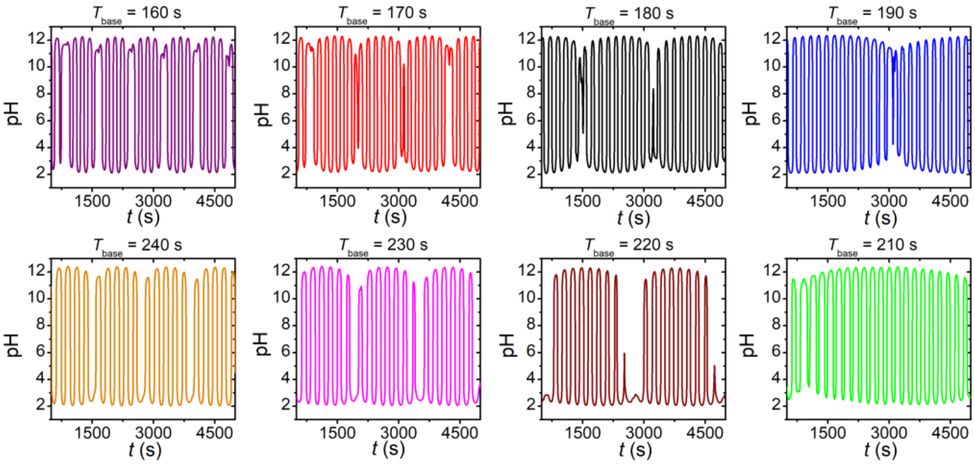

Supplementary Fig. 2 Beat phenomenon in the acid-base neutralization reaction using sinusoidal time-dependent inflow rate functions of the reagents, acid $\left(r(t) = r_0 + r_A \sin\left(\frac{2\pi}{T_{acid}} t\right)\right)$ and alkaline $\left(r(t) = r_0 + r_A \sin\left(\frac{2\pi}{T_{base}} t + \varphi\right)\right)$ solutions with $\varphi = \pi$, r_0 and r_A were 15 μLs^{-1}. The time period of the inflow rate of the acid was fixed, $T_{acid} = 200$ s, and in each experiment T_{base} was varied. The concentrations of the acid and alkaline solutions in the input feed were 0.1 M ($c^0_{H^+} = c^0_{OH^-} = 0.1$ M), respectively.

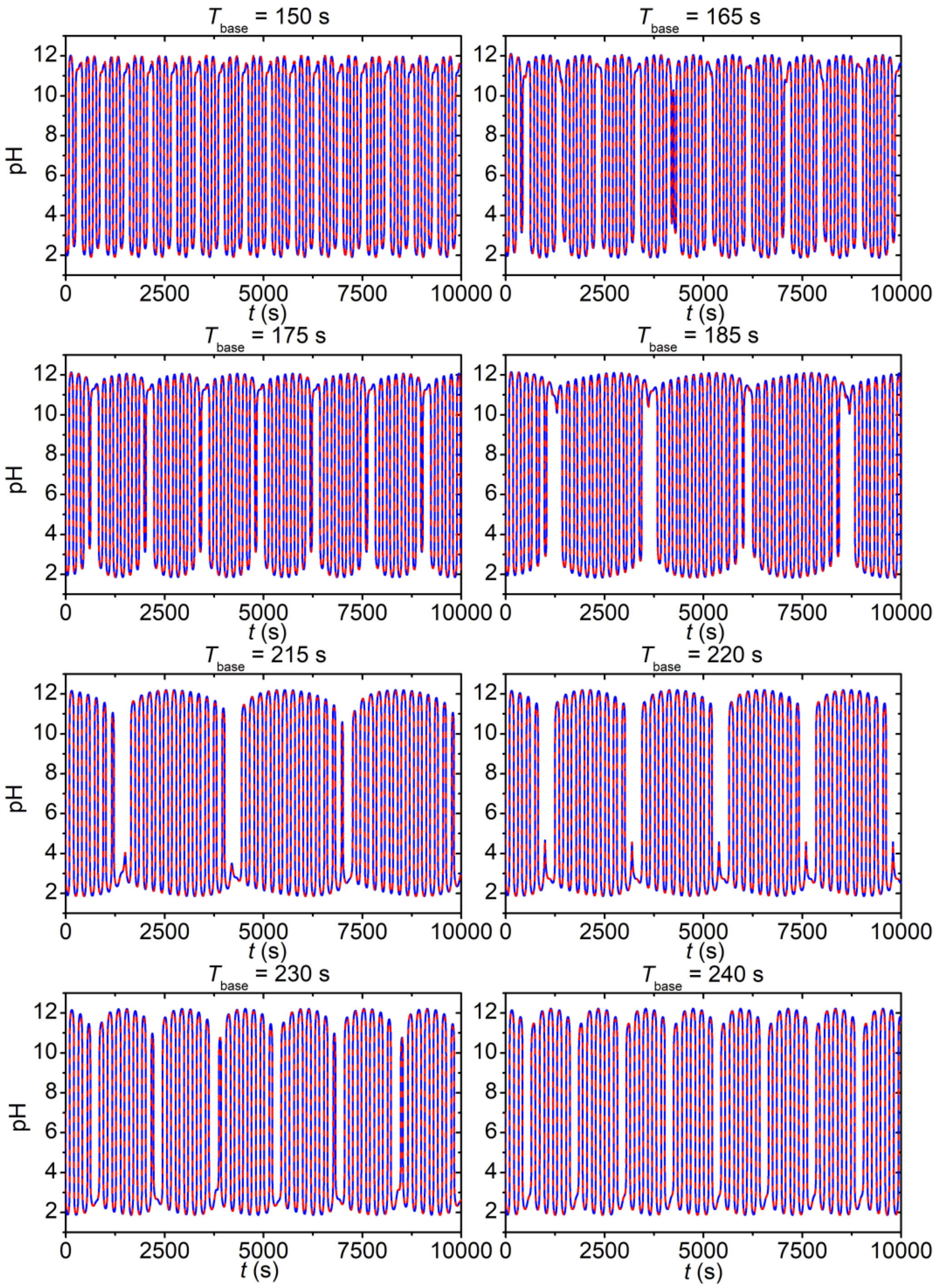

Supplementary Fig. 3 Beat phenomenon in the acid-base neutralization reaction using sinusoidal time-dependent inflow rate functions of the reagents in analytical (solid blue line) and numerical (dashed red line) model, acid $\kappa_{\mathrm{H}^+} = \kappa_0 + \kappa_{\mathrm{A}} \sin\left(\frac{2\pi}{T_{\mathrm{acid}}} t\right)$, and alkaline $\kappa_{\mathrm{OH}^-} = \kappa_0 + \kappa_{\mathrm{A}} \sin\left(\frac{2\pi}{T_{\mathrm{base}}} t + \varphi\right)$ solutions with $\varphi = \pi$, $\kappa_0 = \kappa_{\mathrm{A}} = 2.143 \times 10^{-3}$. The time period of the inflow rate of the acid was fixed, $T_{\mathrm{acid}} = 200$ s, and in each experiment T_{base} was varied. The concentrations of the acid and alkaline solutions in the input feed were 0.1 M ($c^0_{\mathrm{H}^+} = c^0_{\mathrm{OH}^-} = 0.1$ M), respectively.

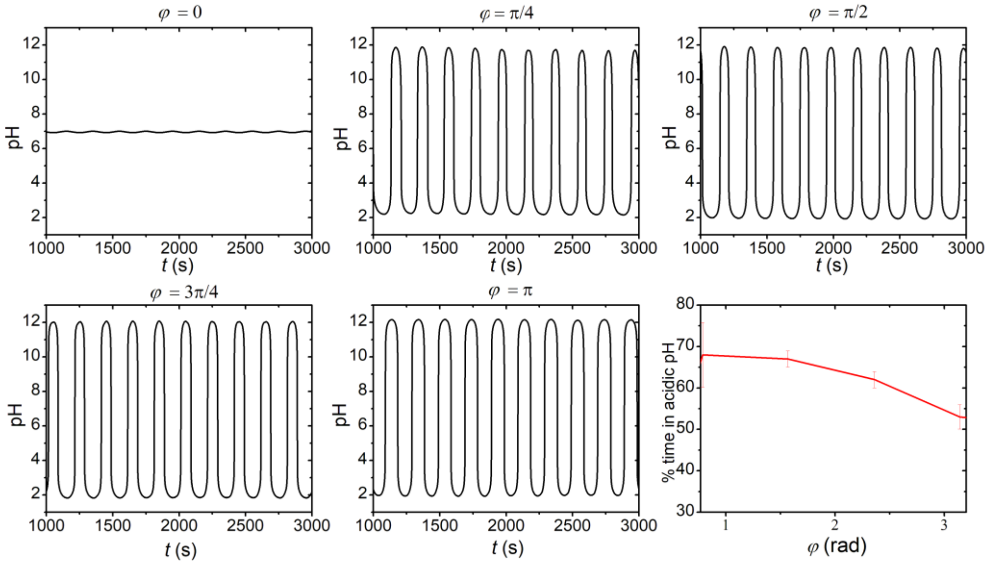

Supplementary Fig. 4 Temporal oscillation patterns in the acid-base neutralization reaction using sinusoidal time-dependent inflow rate functions of the reagents, acid $\left(r(t) = r_0 + r_A \sin\left(\frac{2\pi}{T_{acid}} t\right)\right)$ and alkaline $\left(r(t) = r_0 + r_A \sin\left(\frac{2\pi}{T_{base}} t + \varphi\right)\right)$ solutions using various φ, r_0 and r_A were 15 μLs^{-1} and the dependence of the time spent in the acidic range by the oscillator on the phase difference between the inflow rates of the acid and alkaline solutions. The time period of the inflow rate of the acid and alkaline solutions were fixed, $T_{acid} = T_{base} = 200$ s. The concentrations of the acid and alkaline solutions in the input feed were 0.1 M ($c^0_{H^+} = c^0_{OH^-} = 0.1$ M), respectively.

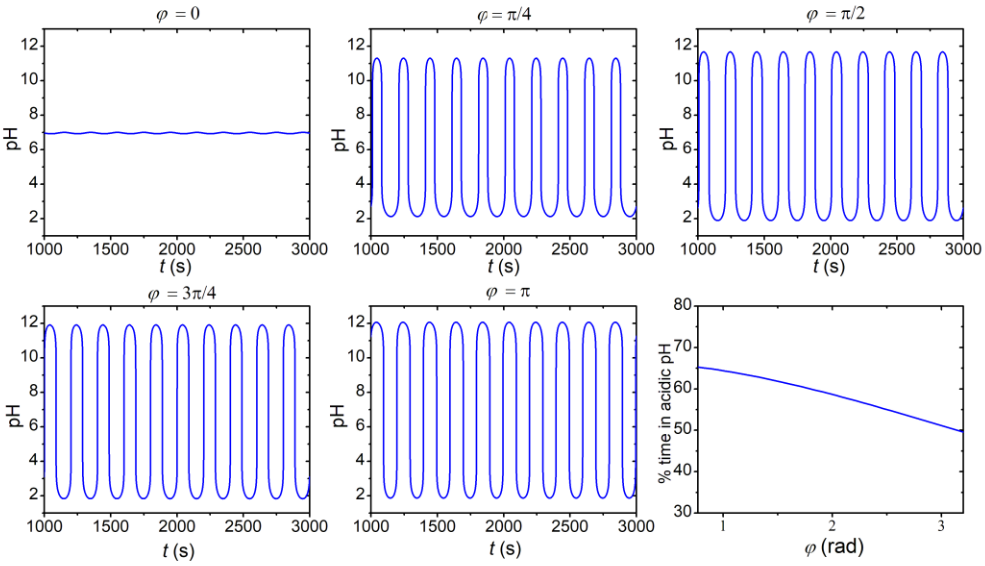

Supplementary Fig. 5 Temporal oscillation patterns in the acid-base neutralization reaction using sinusoidal time-dependent inflow rate functions of the reagents in the numerical model simulations, acid $\kappa_{H^+} = \kappa_0 + \kappa_A \sin\left(\frac{2\pi}{T_{acid}} t\right)$, and alkaline $\kappa_{OH^-} = \kappa_0 + \kappa_A \sin\left(\frac{2\pi}{T_{base}} t + \varphi\right)$ using various φ solutions, $\kappa_0 = \kappa_A = 2.143 \times 10^{-3}$ and the dependence of the time spent in the acidic range by the oscillator on the phase difference between the inflow rates of the acid and alkaline solutions. The time period of the inflow rates of the acid and alkaline solutions were fixed, $T_{acid} = T_{base} = 200$ s. The concentrations of the acid and alkaline solutions in the input feed were 0.1 M ($c^0_{H^+} = c^0_{OH^-} = 0.1$ M), respectively.

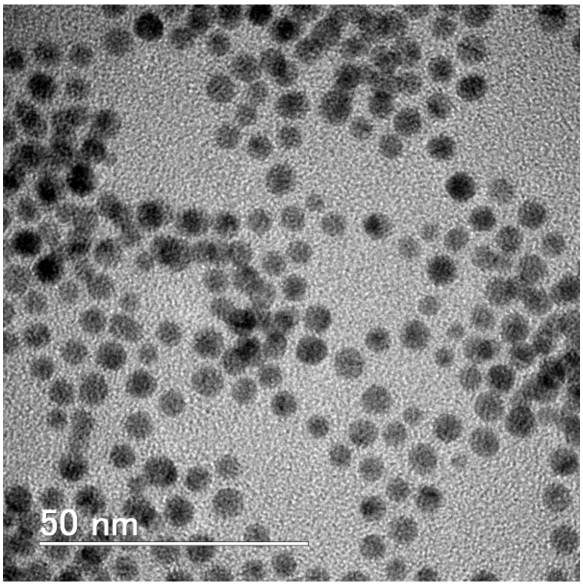

Supplementary Fig. 6 Transmission electron micrograph of the gold nanoparticles used in the experiments.
